# Programmable RNA targeting with clustered regularly interspaced short palindromic repeats (CRISPR) effector Cas7-11 in zebrafish embryos and mammalian cells

**DOI:** 10.1186/s13578-026-01632-w

**Published:** 2026-08-17

**Authors:** Huan Yan, Imtiaz UL Hassan, Kai Yan, Man Yee Cheung, Jiani Deng, Dio Daniel Wuyang, Ke Jiang, Liangji Zhou, Luowei Yuan, Dongqing Cai, Xufeng Qi, Yong Lei, Ping Wei, Hui Zhao

**Affiliations:** 1 Key Laboratory for Regenerative Medicine of the Ministry of Education of China, School of Biomedical Sciences, Faculty of Medicine, The Chinese University of Hong Kong, Shatin, Hong Kong SAR, People’s Republic of China; 2 State Key Laboratory of Genetic Evolution & Animal Models, Kunming Institute of Zoology, Chinese Academy of Sciences, Kunming, 650201 Yunnan China; 3 Department of Obstetrics, The Sixth Affiliated Hospital, Sun Yat-sen University, No. 26 of Yuancun Second Cross Road, Guangzhou, 510655 Guangdong China; 4 Division of Biomedical Health Sciences, School of Medicine, The Chinese University of Hong Kong, Shenzhen, 518172 China; 5 Key Laboratory of Regenerative Medicine of Ministry of Education, Department of Developmental & Regenerative Biology, Jinan University, Guangzhou, 510632 Guangdong China; 6 State Key Laboratory of Quantitative Synthetic Biology, Shenzhen Institute of Synthetic Biology, Shenzhen Institutes of Advanced Technology, Chinese Academy of Sciences, Shenzhen, 518055 China; 7 Kunming Institute of Zoology-The Chinese University of Hong Kong (KIZ-CUHK) Joint Laboratory of Bioresources and Molecular Research of Common Diseases, The Chinese University of Hong Kong, Hong Kong SAR, China; 8 Hong Kong Branch of CAS Center for Excellence in Animal Evolution and Genetics, The Chinese University of Hong Kong, New Territories, Hong Kong SAR, China; 9 Biomedical Sciences Building, The Chinese University of Hong Kong, Hong Kong SAR, People’s Republic of China; 10 Shenzhen University Town, No. 1068, Xueyuan Boulevard, Xil, Nanshan District, Shenzhen, 518055 Guangdong Province People’s Republic of China

**Keywords:** Cas7-11, RNA editing, Knockdown, Zebrafish embryos, Human embryonic stem cells

## Abstract

**Background:**

The CRISPR/Cas7-11 system is a recently characterized RNA targeting tool that exhibits low toxicity, minimal off-target effects, and reduced collateral RNA cleavage. While Cas7-11 has been recently tested in zebrafish embryos as ribonucleoprotein (RNP) complexes, its use as mRNA–single guide RNA (sgRNA) formulations and its application in human embryonic stem cells (hESCs) have not been explored.

**Results:**

In this study, we evaluated the efficiency and specificity of *Cas7-11* in zebrafish embryos and hESCs. In zebrafish,injection ofCas7-11and sgRNA resulted in evident reductions of target transcripts, accompanied by clear phenotypic outcomes. Exogenous*EGFP*mRNA levels were reduced to 31.90%, while endogenous tbxta was reducedto 19.89%, and tyrosinase to 33.18%, respectively, resulting in the no-tail phenotype and reduced pigmentation. Theno-tail phenotype was partially rescued by mRNA overexpression. RNA sequencing confirmed minimal off-target effects. Moreover, Cas7-11 decreased exogenous*Gaussia luciferase*mRNA in human embryonic and HEK293T cells, to 23.52% and to 63.75%, respectively, and correspondingly reduced luciferase activity, without collateral cleavage activity. Targeting pluripotency factor genes, OCT4 and SOX2, in hESCs decreased their mRNA levels and induced differentiation.

**Conclusions:**

Collectively, these findings establish Cas7-11 as a precise and efficient RNA knockdown tool for functional studies in embryonic development and stem cell biology, providing a versatile alternative to DNA-based gene-editing approaches.

**Supplementary Information:**

The online version contains supplementary material available at https://doi.org/10.1186/s13578-026-01632-w.

## Introduction

Current gene-editing studies predominantly use DNA- or RNA-based technologies. RNA editing offers several advantages over traditional DNA editing systems. The most fundamental difference lies in their impact on the genome. DNA editing, typically mediated by technologies such as CRISPR/Cas9, involves permanent modifications to the genomic sequence, which can be inherited by subsequent generations of cells [1]. In contrast, RNA editing is a transient process that modifies post-transcriptional RNA molecules without altering the genomic DNA itself [2]. This reversible nature renders RNA editing particularly advantageous for treating certain genetic diseases, as it allows for correction of specific RNA sequence errors and restoration of normal protein function without introducing permanent genomic changes [2, 3]. Compared to the potentially irreversible side effects associated with DNA editing, RNA editing offers a safer and more flexible therapeutic approach, effectively avoiding genomic off-target effects and unwanted insertions and deletions (indels) resulting from non-homologous end joining (NHEJ) [4, 5]. Furthermore, unlike DNA editing tools, RNA editing does not rely on homology-directed repair (HDR) machinery [6], enabling its application in non-dividing cells. In zebrafish, RNA-mediated gene knockdown circumvents the need for laborious multi-generational genotypic screening to establish stable mutant lines, a process that can be complicated when homozygous mutations affect viability or fertility of maternal founders [7, 8].

While several RNA-targeting tools, such as CRISPR-Cas13d [9], RNA interference (RNAi), and morpholinos [10–18], are currently available, but each is limited by distinct technical limitations. Cas13d, for example, exhibits pronounced collateral cleavage and off-target effects. Conversely, RNAi is ineffective in the zebrafish and *Xenopus* vertebrate models [10–12]. Although widely used in these two models, morpholinos are frequently associated with severe toxicity, off-target binding, and immunogenicity in these same organisms [13–18].

The Cas7-11 system represents a recently characterized RNA-guided RNase that has been shown to exhibit low toxicity and reduced off-target effects in mammalian cell lines [19]. Unlike Cas9, Cas7-11 does not require a protospacer adjacent motif (PAM) sequence at the target site, offering greater targeting flexibility [19, 20]. A recent study evaluated Cas7-11 ribonucleoprotein (RNP) complexes in zebrafish embryos, demonstrating on-target efficacy and minimal collateral activity when targeting endogenous transcripts [21]. However, the application of Cas7-11 delivered as mRNA–single guide RNA (sgRNA) formulations and its application in human embryonic stem cells (hESCs) have not been explored.

In this study, we assessed the ability of Cas7-11 to function as an mRNA-sgRNA system in zebrafish embryos and tested its efficacy for gene knockdown in hESCs, thereby extending its application to previously untested models and delivery modalities [22–25]. Our results demonstrate that Cas7-11 efficiently knocks down RNA expression in both systems, with low off-target propensity, minimal toxicity, and no detectable collateral cleavage activity. Our findings establish Cas7-11 as a robust tool for probing gene function during zebrafish embryonic development and hESC differentiation, thereby expanding the applicability of this novel RNA editing platform. Moreover, the high specificity and safety profile of Cas7-11 underscore its potential for future therapeutic development, particularly for RNA-associated diseases.

## Results

### The Cas7-11 can effectively reduce mRNA expression in zebrafish embryos

**Fig. 1:**
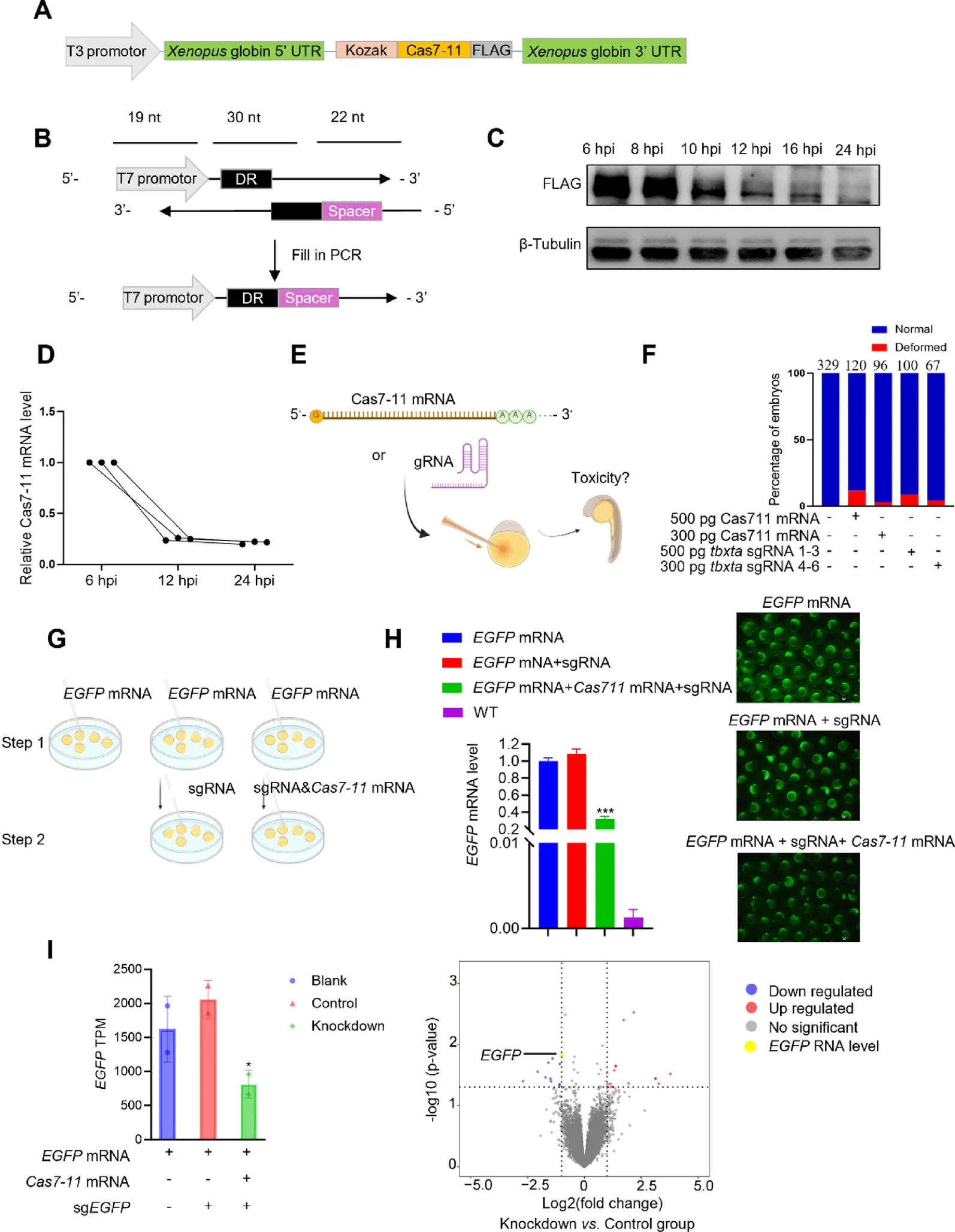
Cas7-11 effectively knocked down exogenous* EGFP* mRNA in zebrafish embryos with low toxicity and minimal off-target effects. **A** Optimized backbone of *Cas7-11* for mRNA transcription used in zebrafish embryos. **B** Schematic diagram showing that the sgRNA was in vitro transcribed from a T7 promoter, containing a direct repeat (DR) for Cas7-11 protein binding and a spacer for target mRNA binding. **C** The Cas7-11 protein was detectable from 6 to 24 h post-injection following injection of *Cas7-11 2×FLAG* mRNA (500 pg/embryo). **D** RT-qPCR indicated the relative level of injected *Cas7-11* mRNA (500 pg/embryo), normalized to the level at 6 h after injection. **E**,** F** Toxicity evaluation of Cas7-11 mRNA and sgRNA in zebrafish embryo. Dose-dependent deformation rates in zebrafish embryos injected at the 1–2 cell stage with either Cas7-11 mRNA or sgRNA. Reducing the injection dosage from 500 pg to 300 pg per embryo substantially decreased deformation rates for both Cas7-11 mRNA (from 11.6% to 3.1%) and sgRNA (from 9.0% to 4.3%). **G**,** H** Cas7-11-mediated *EGFP* knockdown efficiency. The experimental design is shown in G. After 8 h post-injection, the EGFP fluorescence signal in the experimental group decreased markedly compared with the blank and control groups. RT-qPCR showed that the mRNA in the experimental group (green bar) showed a substantial decrease to 31.90% compared to the blank group (blue bar), while the control group remained almost unchanged (108.78%) compared to the blank group (H). Data are presented as mean ± SE from two independent experiments (three biological replicates per experiment, *n* = 20 embryos/replicate). ***P < 0.001 (Student’s t-test). *cdk2ap* mRNA was used as the internal standard control. **I** Volcano plots showing fold changes and p-values of gene expression in the comparison between the experimental group and the control group. Two biological repeats at 6 hpi were employed for RNA-Seq analysis. The up-regulated gene is shown in red, while the down-regulated gene is marked in blue

To assess the functional capacity of Cas7-11 in zebrafish, we first subcloned the *Cas7-11* coding sequence into the *pCS2 +* vector. However, western blot analysis of embryos injected with mRNA transcribed from this construct could not detect Cas7-11 protein expression (Supplementary Figure S1B). To improve translation efficiency, we introduced a *Kozak* sequence (GCCGCCATGG) at the start of the *Cas7-11* open reading frame and flanked the coding sequence with *Xenopus globin* 5′-UTR and 3′-UTR elements. A 2× FLAG tag was inserted in-frame at the C-terminus to facilitate protein detection. The resulting optimized construct was then subcloned into the *pCS2 +* vector for in vitro transcription (Fig. 1A).

The in vitro-transcribed mRNA encoding the modified Cas7-11 was injected into zebrafish embryos at the 1- or 2-cell stage. Western blot analysis confirmed Cas7-11-FLAG protein expression from 6 to 24 h post-injection (hpi) (Fig. 1C), consistent with the decay profile of the injected mRNA. Quantitative RT-PCR (RT-qPCR) showed that *Cas7-11* mRNA levels decreased to 24.77% at 12 hpi and 21.22% at 24 hpi, relative to levels at 6 hpi (Fig. 1D).

Cas7-11 recognizes and binds to the direct repeat (DR) sequences of sgRNAs [19]. sgRNAs were synthesized as illustrated in Fig. 1B. Spacer sequences with a length of 22 nt were designed to be reverse complementary to target mRNA regions, such as the coding sequence (CDS) or 5′ UTR, which are known to elicit robust phenotypic changes. To optimize cleavage efficiency, target accessibility was predicted using the RNAfold web server. The sgRNA sequence was selected based on low base-pairing probability from minimum free energy predictions. We designed at least three sgRNAs for each gene, spaced at least 50 nucleotides apart to minimize competitive binding.

To assess potential toxicity, zebrafish embryos at the 1-2-cell stage were injected with varying doses of *Cas7-11* mRNA and sgRNA (Fig. 1E). Injection of 500 pg of *Cas7-11* mRNA per embryo resulted in 11.6% deformed embryos (14 out of 120), whereas 300 pg resulted in 3.1% deformation (3 out of 96). Likewise, 500 pg of sgRNA per embryo caused 9% deformation (9 out of 100), compared with 4.4% at 300 pg (3 out of 67) (Fig. 1F). These results indicate a dose-dependent toxicity for both components.

We next assessed whether Cas7-11 could target and reduce exogenous mRNA expression in vivo. Embryos at the 1–2 cell stage were divided into three groups, each receiving equal amounts of *EGFP* mRNA. The experimental group was co-injected with *Cas7-11* mRNA, gene-specific sgRNA, and *EGFP* mRNA; the control group received sgRNA and *EGFP* mRNA; and the blank group received *EGFP* mRNA only (Fig. 1G). Fluorescence imaging showed a marked reduction in EGFP signal in the experimental group, while control and blank groups displayed comparable fluorescence (Fig. 1H). Consistent with this observation, RT-qPCR confirmed that *EGFP* mRNA levels in the experimental group decreased to 31.90% of those in the blank group, while no significant difference was detected between the control and blank groups (Fig. 1H). RNA-seq analysis further validated the specific reduction of *EGFP* mRNA in the experimental group. Based on the volcano plot analysis, aside from the specific knockdown of *EGFP*, only a small number of genes exhibited differential expression. These non-specifically dysregulated genes were found to be associated with the protein-motoring process, an effect likely attributable to the cellular response to translating *Cas7-11* mRNA into protein following its injection into zebrafish embryos (Fig. 1I). To validate RNA-seq analysis, we selected seven genes that showed no significant changes in the RNA-seq volcano plot and examined their expression using qRT-PCR. No significant differences in the expression of these genes were detected among embryos injected with either *EGFP* mRNA, *EGFP* mRNA+sgRNA, or *EGFP* mRNA+*Cas7-11* mRNA+sgRNA (Figure S3). Overall, these data demonstrate the high fidelity and low off-target effects of the Cas7-11 system.

**Fig. 2:**
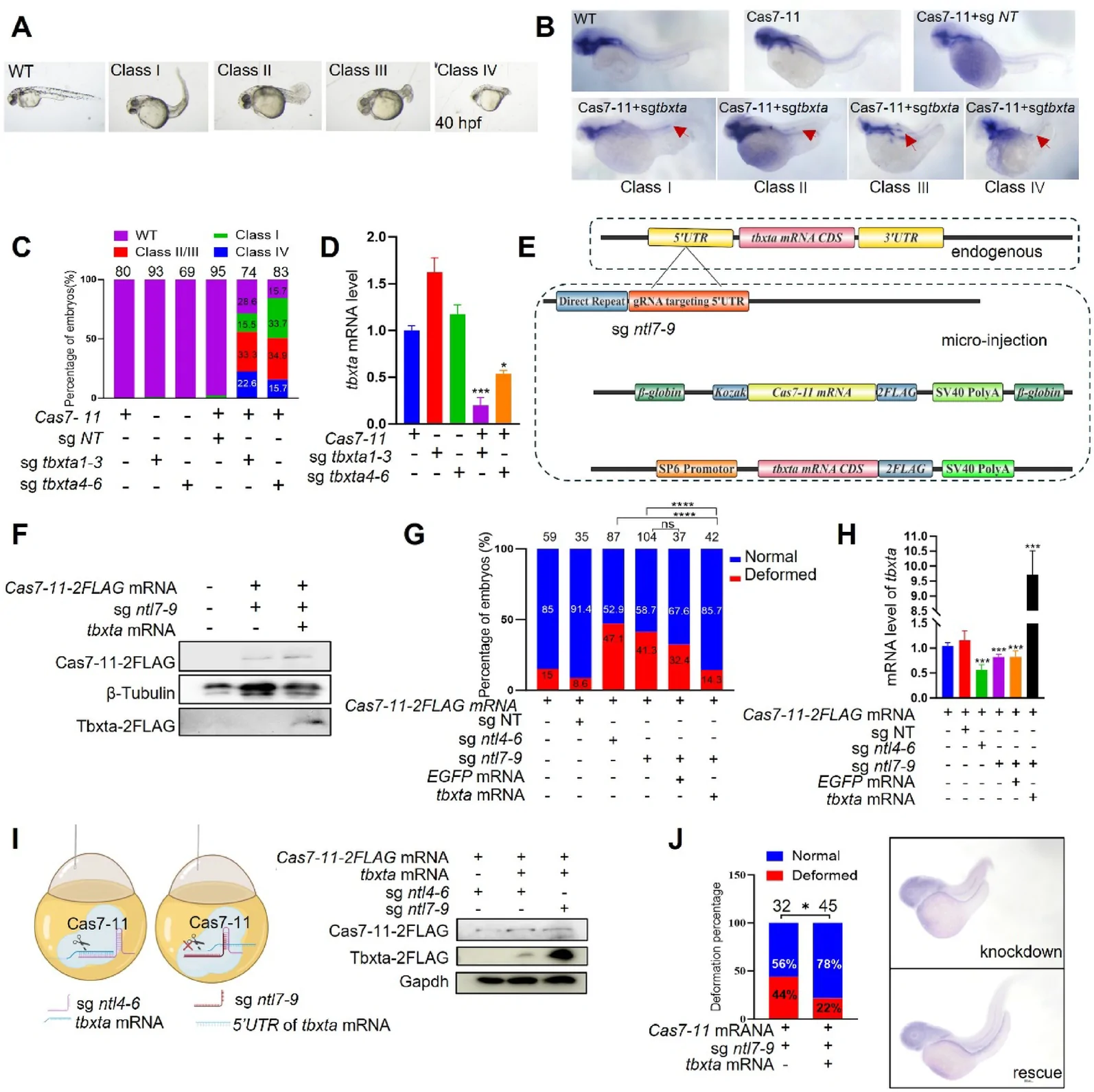
Cas7-11 specifically knocked down the* tbxta* mRNA with a no-tail phenotype. **A** Representative images (40 hpf) of anteroposterior axis deformities induced by *Cas7-11* mRNA (300 pg) and sgRNA (600 pg) targeting *tbxta* mRNA. Phenotypes are classified by severity into four categories. Class I: short tail (least extreme). Classes II and III: absence of notochord and short tail (medium level). Class IV: absence of a notochord and an extremely short tail (most extreme). Scale bar, 0.5 mm. **B** Whole-mount in *situ* hybridization (WISH) for the notochord marker gene *shh* in embryos either uninjected or injected with *Cas7-11* mRNA (300 pg/embryo) and *tbxta* sgRNA (600 pg/embryo). **C** Quantification of the induced phenotypes by different *tbxta* sgRNA sets. **D** RT-qPCR analysis showed the levels of *tbxta* mRNA at 6 hpi in embryos injected with the indicated components. *cdk2ap* mRNA was used as the normalization control. **E** Schematic diagram showing sgntl7-9 targeting the *tbxta* 5’UTR, and the constructs used for Cas7-11 expression and rescue of no-tail phenotype. **F** Western blot confirming the expression of Tbxta-FLAG protein in the rescue group at 8 hpi. **G** Quantification of the no-tail phenotype rescue. Stacked bar plots displayed the percentage of observed phenotypes using *no tail* (*ntl 7–9*) sgRNA targeting *5’UTR* (300 pg/embryo) or *no tail* (*ntl 4–6*) sgRNA targeting coding sequence (*CDS*) (300 pg/embryo) and *Cas7-11-2×FLAG* mRNA (100 pg/embryo) co-injected with different mRNAs: *EGFP* mRNA (100 pg/embryo) as a control, *CDS tbxta-2×FLAG* mRNA (100 pg/embryo) as rescue treatment. As an additional control, an unrelated sgNT was included (300 pg/embryo). The number of embryos evaluated (n) was shown for each condition. The stacked bar plots illustrated the percentage of the observed phenotype (red bar) under the various injection conditions. Chi-square tests were employed to analyze difference between the rescue group and *ntl* disruption group. **H** RT-qPCR analysis showed the levels of *tbxta* mRNA at 6 hpi in embryos injected with the indicated components. **I** Western blot demonstrating the reduction of Tbxta-2*×*FLAG protein in experimental knockdown embryos compared to controls at 8 hpi. **J** WISH and quantification of the *shh* expression induced by different *tbxta s*gRNA sets, showing partial restoration of notochord formation in *tbxta* mRNA-rescued embryos. The number of embryos with normal morphology and *shh* expression and those with deformed morphology and abnormal *shh* expression was counted for the bar chart and analyzed by the chi-square test

We next applied the Cas7-11 system to knock down the endogenous *tbxta* gene, which is essential for tail formation in zebrafish [26, 27]. Loss of *tbxta* function leads to a no-tail phenotype and disrupted expression of *sonic hedgehog* (*shh*), a marker of notochord development [26–29]. Embryos co-injected with *Cas7-11* mRNA and a pool of three *tbxta*-targeting sgRNAs exhibited a no-tail phenotype (Fig. 2A). RT-qPCR showed that *tbxta* mRNA levels decreased to 19.89% in embryos injected with *Cas7-11* mRNA and the mixture of sgRNA 1–3, to 53.73% with *Cas7-11* mRNA and sgRNA 4–6 (Fig. 2D). Phenotypic severity was categorized into four classes. Class I is defined as the short tail, which is the least extreme. Classes II and III are defined as the absence of a notochord and a short tail, which is of medium level. Class IV is defined as the absence of a notochord and an extremely short tail, which is the most extreme. Embryos injected with *Cas7-11* mRNA and sgRNA 1–3 displayed 22.62% Class IV (most severe), 33.33% Class II/III (moderate), and 15% Class I (mild) phenotypes. In the *Cas7-11* mRNA and sgRNA 4–6 co-injection group, 15.66% exhibited Class IV, 34.94% Class II/III, and 33.73% Class I phenotypes. Control injections with sgRNA alone showed minimal or no deformity (Fig. 2C). Whole-mount in situ hybridization for *shh* revealed disrupted expression patterns in the experimental group, while controls retained normal *shh* signals (Fig. 2B).

To assess specificity, we tested whether the observed phenotype could be rescued by co-injection of *tbxta* mRNA lacking the 5′ UTR target sequence. sgRNAs were designed to target the endogenous 5′ UTR, leaving the exogenous coding sequence intact (Fig. 2E). We first observed that the overexpressed *tbxta* mRNA could be translated into Tbxta-2FLAG protein in embryos (Fig. 2F). The rescue group, co-injected with *Cas7-11* mRNA, 5′ UTR-targeting sgRNA (sg *ntl7–9*), and *tbxta* CDS mRNA, showed a markedly reduced no-tail phenotype (14.3%) compared to knockdown groups (47.1% for CDS-targeting using sg *ntl4-6*, and 41.3% for UTR-targeting), with a significant difference. The rescue control group (injected with *EGFP* mRNA) showed 32.4% deformity without a significant difference compared to the knockdown group, while the blank group (*Cas7-11* mRNA only) exhibited 15%. We further labeled the embryos co-injected with *Cas7-11* mRNA and sgNT as the knockdown control group, which lacks targeting sites in zebrafish transcripts, and observed only 8.6% deformity (Fig. 2G). RT-qPCR confirmed a 9.71-fold increase in *tbxta* mRNA in the rescue group relative to the blank group, indicating successful overexpression. In addition, the *tbxta* mRNA level in the knockdown control group did not differ significantly from that in the blank group. However, it decreased to 56.41% in the knockdown group targeting the CDS and to 82.06% in the knockdown group targeting the 5’UTR (Fig. 2H). Western blot confirmed the reduction of exogenous Tbxta protein in embryos co-injected with CDS-targeting sgRNA, indicating effective knockdown of the introduced mRNA (Fig. 2I). In situ hybridization for *shh* revealed abnormal expression in 44% of knockdown embryos, compared to 22% in the rescue group (Fig. 2J). These results indicated that the addition of *tbxta* could partially rescue the no-tail phenotype and *shh* expression.

**Fig. 3:**
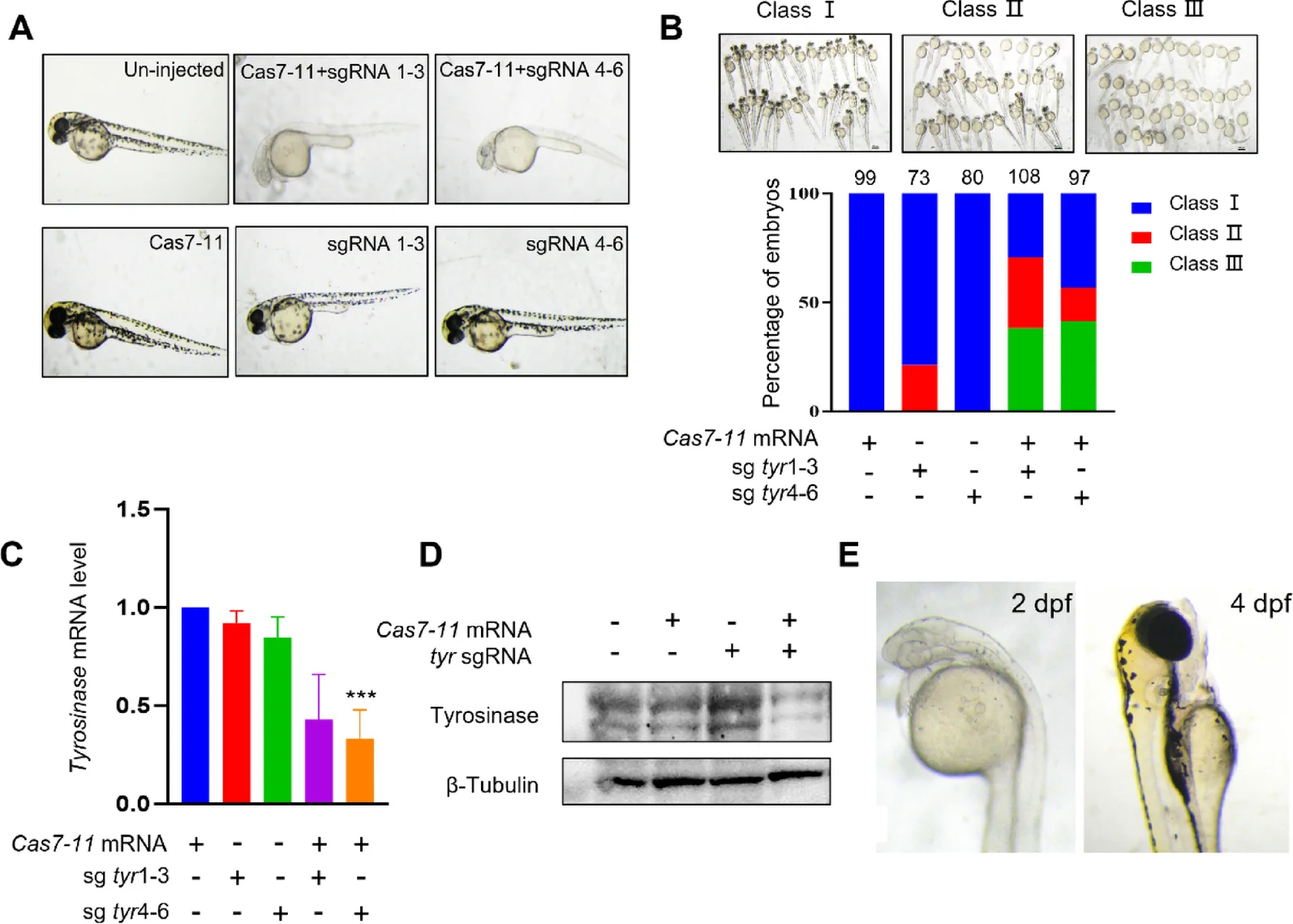
The Cas7-11 system effectively knocked down *tyrosinase* in zebrafish embryos. **A** Reduction of pigmentation induced by knockdown of *tyrosinase* by Cas7-11. No obvious decrease in pigmentation in embryos injected alone with *Cas7-11* mRNA or sgRNA. A significant depigmentation phenotype was induced by co-injecting Cas7-11 mRNA and an sgRNA targeting the *tyrosinase* gene. **B** Quantification of the phenotypes (60 hpi) induced by either Cas7-11 mRNA, sgRNA, and a combination of *Cas7-11* and sgRNA as indicated. Class I: weaker pigmentation in eyes and bodies than wide-type phenotype embryos (least extreme). Class II: absence of pigmentation within eyes or bodies (medium level). Class III: absence of pigmentation within eyes and bodies (most extreme). Targeting different *tyrosinase* mRNA sites with the Cas7-11 system recapitulated the no-pigmentation phenotype. Stacked bar plots showed the percentage of observed phenotypes under various injection conditions. **C** RT-qPCR showed *tyrosinase* expression at 32 hpi in the embryos injected with the indicated combinations. *cdk2ap* was used as the internal standard control. **D** Embryos were injected with either *Cas7-11* mRNA, sgRNA, or a combination of mRNA and sgRNA as indicated. After 22 h, embryos were lysed and probed with Tyrosinase and β-Tubulin antibodies. Tyrosinase protein was decreased in *Cas7-11* mRNA and sgRNA co-injected embryos compared to the WT and *Cas7-11* mRNA or sgRNA injection-only groups. **E** Pigmentation in zebrafish returned to normal on day 4 post-fertilization, while it displayed depigmentation at day 2 post-fertilization

To distinguish between specific knockdown effects and general toxicity, we targeted the *tyrosinase* gene, which encodes the tyrosinase enzyme for catalyzing the first step of melanin production. Depletion of *tyrosinase* expression results in a transient depigmentation phenotype clearly distinguishable from morphological abnormalities [30]. Embryos co-injected with *Cas7-11* mRNA and *tyrosinase*-targeting sgRNA showed significant depigmentation (Fig. 3A). Phenotypic severity was classified into three categories. Class I, the mildest category, is defined as weaker pigmentation in the eyes and bodies than in wild-type embryos. Class II, representing the intermediate severity, is characterized by the absence of pigmentation either in the eyes or body. Class III, the most severe category, was defined by the complete absence of pigmentation in both the eyes and the body. Co-injection of *Cas7-11* with sgRNA 1–3 resulted in 38.2% Class III and 32.7% Class II phenotypes, whereas co-injection with sgRNA 4–6 resulted in 41.4% Class III and 15.4% Class II phenotypes. In contrast, control groups injected with sgRNA alone or *Cas7-11* alone exhibited milder or no depigmentation (Fig. 3B). Consistent with the phenotype, RT-qPCR confirmed *tyrosinase* mRNA level was decreased to 33.18% in the co-injection group (Fig. 3C), and western blot showed corresponding protein reduction (Fig. 3D). Notably, pigmentation recovered by 4 days post-fertilization, indicating that Cas7-11 mediates transient gene knockdown (Fig. 3E).

These results demonstrate that the Cas7-11 system can efficiently and specifically reduce both exogenous and endogenous mRNA levels in zebrafish embryos, with minimal off-target effects and a transient phenotype that is well suited for developmental studies.

### The Cas7-11 system can knock down gene expression in mammalian cells

**Fig. 4:**
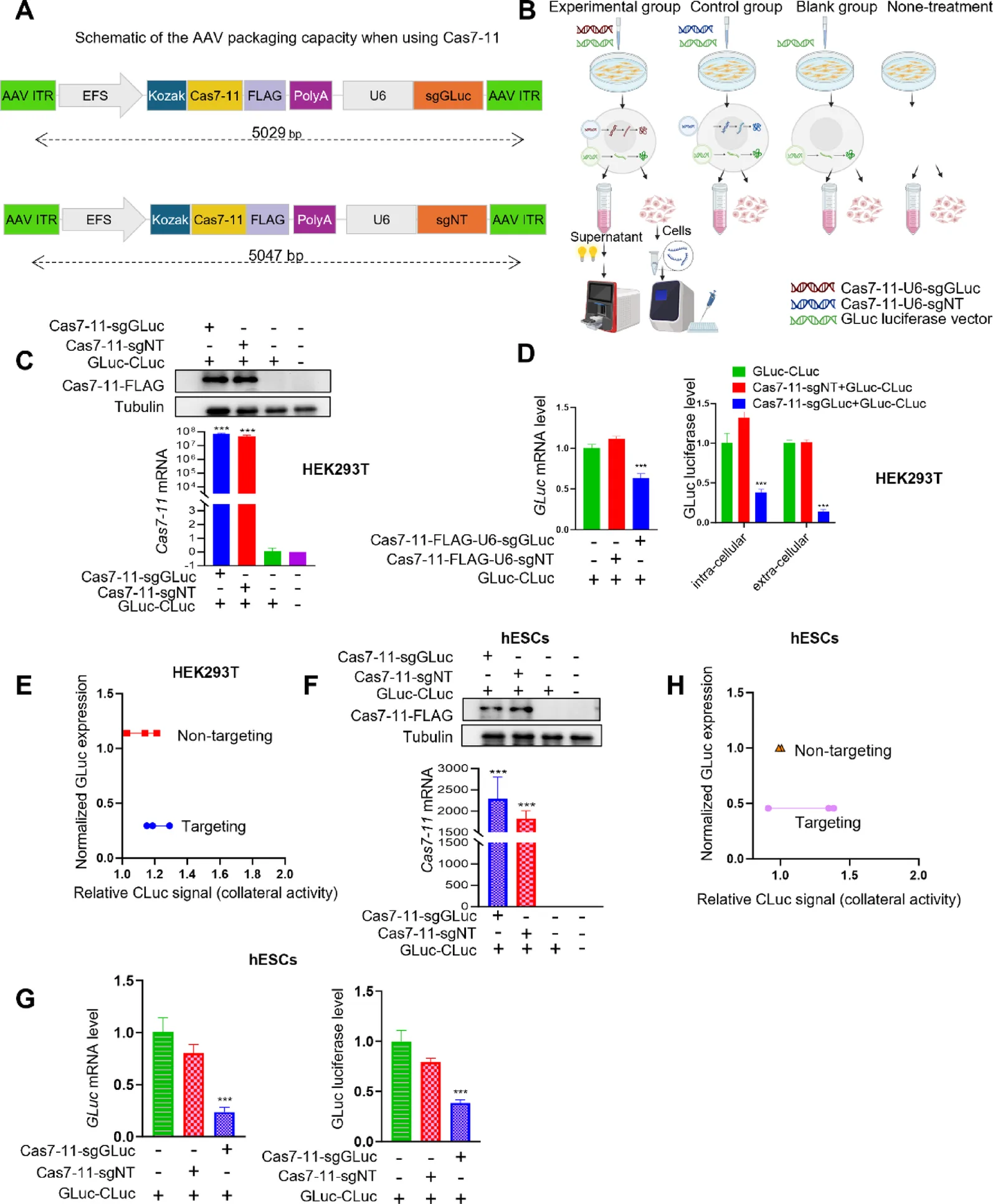
Cas7-11 knocked down the exogenous *GLuc* mRNA in HEK293T cells and hESCs. **A** Schematic of AAV constructs Cas7-11-2FLAG and the sgRNA targeting *GLuc* (or a non-targeting control, sgNT). **B** Experimental workflow for Cas7-11 delivery and the *GLuc*-*CLuc* dual-luciferase reporter assay. **C** Validation of Cas7-11 expression in HEK293T cells by Western blot (FLAG) and RT-qPCR (*GAPDH* internal control) 48 h post-AAV transduction. **D** Quantification of *GLuc* knockdown in HEK293T cells via RT-qPCR and luciferase activity assays. *GLuc* levels were normalized to the *CLuc* internal control. **E** Assessment of collateral cleavage in HEK293T cells, showing specific reduction of *GLuc* without significant downregulation of the co-expressed *CLuc* reporter. **F** Validation of Cas7-11 expression in H1 hESCs by Western blot and RT-qPCR 48 h post-plasmid transfection. *GAPDH* mRNA served as the internal standard control. *Cas7-11* mRNA exhibited significantly higher expression in both the experimental and control groups compared to the blank group and non-treated cells. **G** Quantification of *GLuc* knockdown in hESCs via RT-qPCR and luciferase activity assays, normalized to *CLuc*. Statistical significance was assessed using a Student’s t-test (***P < 0.001). **H** Assessment of collateral cleavage in hESCs, confirming specific *GLuc* targeting without affecting *CLuc* levels. (*n* = 3)

We next evaluated whether the Cas7-11 system could mediate gene knockdown in mammalian cells, particularly in human embryonic stem cells (hESCs). To identify an optimal configuration for Cas7-11 expression in mammalian cells, we cloned various versions of the *Cas7-11* sequence into the *pCS2* + vector (Supplementary Figure S1A). Among the constructs tested, *pCS2-Kozak-Cas7-11* with a C-terminal 2×*FLAG* tag yielded the highest Cas7-11 protein expression (Supplementary Figure S1A). This optimized *Kozak-Cas7-11-*2×*FLAG* sequence was subsequently cloned into an adeno-associated virus (AAV) backbone, along with a sgRNA targeting the exogenous *Gaussia luciferase* (*GLuc*) mRNA. A non-targeting sgRNA (sgNT) was included as a specificity control (Fig. 4A).

To assess knockdown efficiency, we employed a dual-luciferase reporter system in which *GLuc* and *Cypridina* luciferase (*CLuc*) were co-expressed from a single plasmid. Both are secreted luciferases, enabling measurement of protein levels from cell supernatants, while mRNA levels were quantified from cell lysates (Fig. 4B). CLuc served as an internal control for normalization of both luciferase activity and transcript abundance.

HEK293T cells were infected with AAV-Cas7-11 particles at a titer of 2 × 10^9^ vg/mL. Cas7-11-2×FLAG protein can be detected at 48 h post-infection (Fig. 4C). RT-qPCR revealed substantial increases in Cas7-11 mRNA levels, 6.87 × 10^7^-fold in cells infected with the Cas7-11-sgGLuc system (experimental group) and 4.88 × 10⁷-fold in cells infected with the Cas7-11-sgNT system (control group), relative to non-treated cells (Fig. 4C). *GLuc* mRNA levels in the experimental group decreased to 63.75% of those in the blank group (transfected with the dual-luciferase plasmid alone), indicating successful target knockdown (Fig. 4D). Consistent with mRNA reduction, GLuc luciferase activity in the experimental group declined to 38.37% intracellularly and 14.33% extracellularly compared to the blank group (Fig. 4D). To assess potential collateral cleavage, we measured CLuc activity, which was co-expressed with GLuc from the same transcript. No significant difference in CLuc expression was observed between the targeting and non-targeting groups, indicating that Cas7-11 selectively cleaved *GLuc* mRNA without affecting the neighboring coding sequence (Fig. 4E).

We next tested the Cas7-11 system in hESCs. Cells were infected with the Cas7-11-sgGLuc (experimental group), Cas7-11-sgNT (control group) system, or treated with the dual-luciferase plasmid alone (blank group). Western blot confirmed Cas7-11-2×FLAG expression at 48 h post-treatment in both virus-infected groups (Fig. 4F). RT-qPCR showed that Cas7-11 mRNA levels were significantly increased in both the experimental and control groups, reaching 2,293.85-fold and 1,824.75-fold higher levels, respectively, than in untreated hESCs (Fig. 4F). *GLuc* mRNA in the experimental group was reduced to 23.52% of that in the blank group, indicating effective knockdown (Fig. 4G). Accordingly, extracellular GLuc luciferase activity decreased to 38.35% in the experimental group relative to the blank group, and was significantly lower than in the control group (Fig. 4G). To assess potential collateral cleavage, we measured CLuc activity, which was co-expressed with GLuc from the same transcript. No significant difference in CLuc expression was observed between the targeting and non-targeting groups, indicating that Cas7-11 selectively cleaved *GLuc* mRNA without affecting the neighboring coding sequence in hESCs (Fig. 4H).

**Fig. 5:**
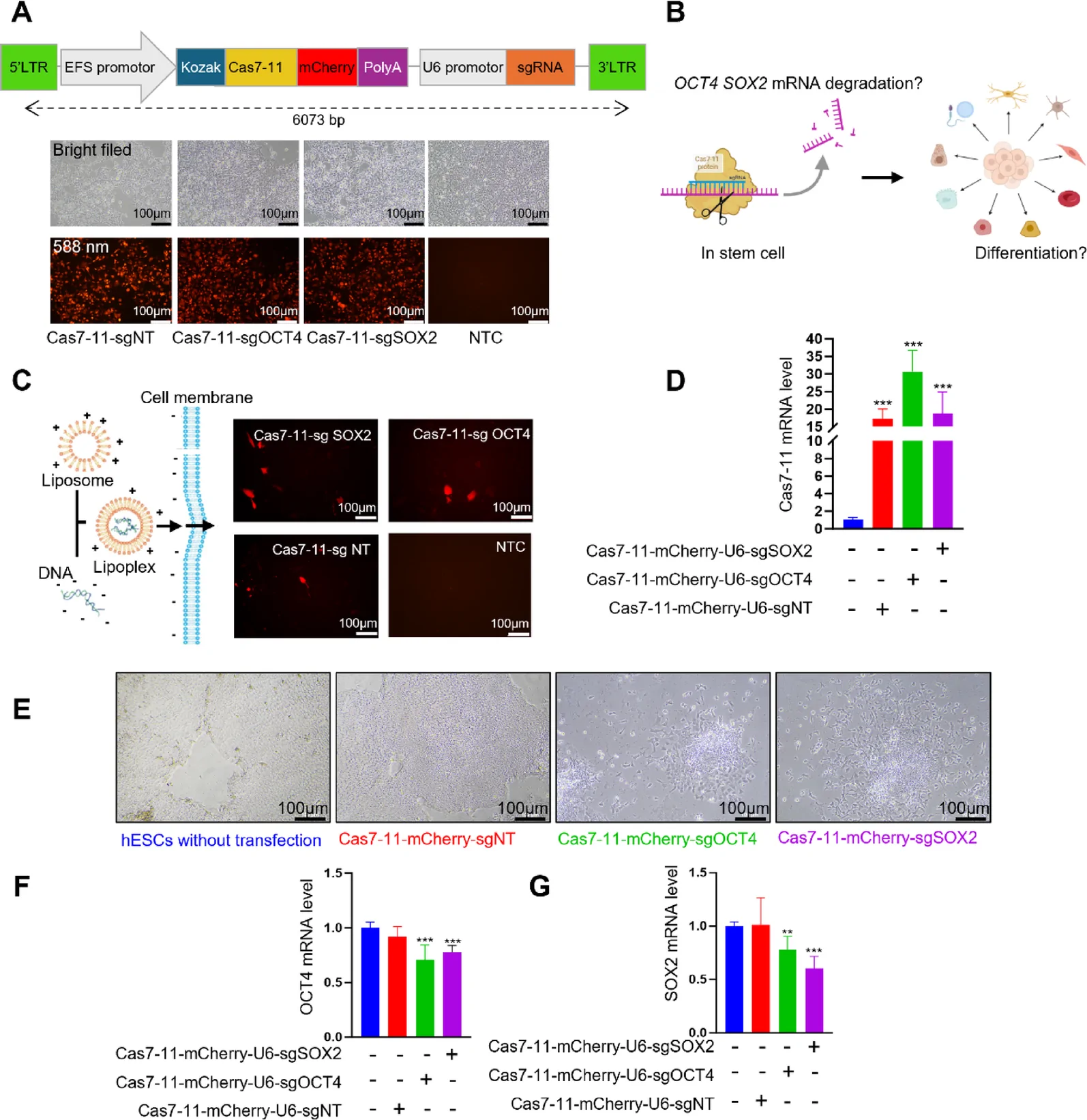
Cas7-11 knocked down the endogenous SOX2 and OCT4 in hESCs. **A** Schematic of lentiviral construct encoding Cas7-11-mCherry and sgRNA targeting *OCT4*, *SOX2*, or a non-targeting control (sgNT), alongside fluorescence microscopy confirming expression in HEK293T cells at 48 h post-transfection (scale bar, 0.1 mm). **B** Schematic diagram showing the predicted consequence of knockdown of *SOX2* and *OCT4* by the Cas7-11, leading to loss of pluripotency and induction of differentiation. **C** Fluorescence microscopy confirming successful liposome-mediated delivery and expression of Cas7-11-mCherry in hESCs (scale bar, 0.1 mm). **D** RT-qPCR showed *Cas7-11* mRNA expression in the transfected hESCs. *GAPDH* mRNA was used as the internal standard control. **E** Morphological assessment of hESCs 4 days post-transfection. Cells targeted with sgOCT4 or sgSOX2 exhibit loss of stem cell colony formation and undergo differentiation, whereas sgNT controls maintain normal morphology (scale bar, 0.1 mm). Human embryonic stem cells were treated with the indicated plasmid for 4 days. The cells were seeded in a 6-well plate at 3 × 10^5^. The cells in each well were treated with 1 µg of the *Cas7-11-mCherry-U6-sgRNA* plasmid. The *OCT4*- and *SOX2*-targeted stem cells showed no cluster formation, and the cells began to differentiate. While the *Cas7-11-sgNT*-transfected hESCs showed no significant difference in morphology compared with the non-treated cells (scale bar, 0.1 mm). **F**,** G** RT-qPCR quantification of (F) *OCT4* and (G) *SOX2* mRNA knockdown efficiencies in hESCs following Cas7-11 targeting

We then examined whether Cas7-11 could knock down endogenous genes in hESCs, targeting the pluripotency markers *OCT4* and *SOX2*. Both genes are essential for maintaining stem cell identity, and knockdown of the two genes is expected to induce differentiation [31, 32] (Fig. 5B). To facilitate monitoring of Cas7-11 expression, the Cas7-11 construct was engineered to include an mCherry reporter (Fig. 5A). mCherry fluorescence was observed in hESCs following transfection, indicating robust Cas7-11 expression (Fig. 5C). RT-qPCR showed that *Cas7-11* expression was increased by 30.74-fold, 18.82-fold, and 17.32-fold in cells treated with Cas7-11-sgOCT4, Cas7-11-sgSOX2, and Cas7-11-sgNT system, respectively, compared to untreated cells (Fig. 5D).

Morphological changes were evident by day 4 post-treatment. While non-treated and sgNT-targeted hESCs retained typical stem cell morphology, cells targeted with the Cas7-11-sgOCT4 or Cas7-11-sgSOX2 system exhibited loss of pluripotency characteristics and signs of differentiation (Fig. 5E). In cells targeted with Cas7-11-sgOCT4, *OCT4* mRNA decreased to 70.87% of non-treated levels at 48 h, whereas sgNT-targeting controls showed no significant change (Fig. 5F). Given the known regulatory interplay between *OCT4* and *SOX2* [33], we also examined *SOX2* expression following *OCT4* knockdown. *SOX2* mRNA levels were reduced to 77.66% in the sgOCT4-targeted group (Fig. 5F). Conversely, targeting *SOX2* with the Cas7-11-sgSOX2 system reduced *SOX2* mRNA to 60.30% of non-treated levels and led to a concomitant decrease in *OCT4* mRNA to 77.84% (Fig. 5G). These results confirm that Cas7-11 can effectively knock down endogenous genes in hESCs and that the resulting perturbations reflect the known regulatory relationships among pluripotency factors.

## Discussion

In this study, we evaluated the Cas7-11 system in zebrafish embryos, hESCs, and HEK293T cells, broadening the utility of this RNA-targeting platform across new biological models and delivery modalities. RNA sequencing analysis confirmed low off-target effects, supporting the precision of this editing platform. Consistent with previous reports [10, 13–19], Cas7-11 exhibited no apparent collateral cleavage activity in our experimental settings and showed a favorable safety profile. The high knockdown efficiency observed in zebrafish demonstrates that Cas7-11 can function effectively within the complex macromolecular environment of a developing organism, overcoming barriers that are not recapitulated in vitro. Therefore, it may serve as an alternative gene knockdown approach to morpholinos in zebrafish. Moreover, the high efficacy of gene knockdown by Cas7-11 in mammalian cell lines supports the compatibility of this RNA-targeting machinery with the mammalian transcriptome.

We observed slightly higher editing efficiency for *tbxta* mRNA compared to *tyrosinase* mRNA in zebrafish embryos under identical injection conditions. This difference may be attributed to the temporal expression patterns of the target genes. The *tyrosinase* is expressed later (approximately 16.5 h post-fertilization, hpf) than *tbxta* (4–5 hpf) during development [30, 34, 35]. Given that Cas7-11 protein expression peaks within a limited window (6–24 h post-injection), the earlier-expressing *tbxta* may be more efficiently targeted. The rapid developmental timeline of zebrafish embryos further narrows the editing window, highlighting the importance of aligning target gene expression with Cas7-11 activity. This observation is consistent with recent findings in the optimized RfxCas13d system, where targeting of mid- to late-zygotically transcribed genes also required enhanced delivery strategies to achieve efficient knockdown [21].

Since its initial characterization, Cas7-11 has been primarily studied in mammalian cell lines using constitutive expression systems [19]. A recent study applied Cas7-11 to zebrafish embryos as RNP complexes, achieving efficient mRNA knockdown without collateral activity [21]. Here, we extend these findings in several important ways. First, we demonstrate that Cas7-11 delivered as mRNA–sgRNA, rather than pre-assembled RNP, is functional in zebrafish embryos, offering a potentially more scalable and flexible delivery format. Second, we provide the first evidence that Cas7-11 can achieve effective gene knockdown in human embryonic stem cells, targeting both exogenous reporters and endogenous pluripotency genes, *OCT4* and *SOX2*, to induce differentiation. This establishes Cas7-11 as a versatile tool not only for developmental biology but also for stem cell research, a field not addressed in previous studies. Third, we demonstrate AAV-mediated delivery of Cas7-11 in mammalian cells, highlighting its potential for therapeutic applications. While the on-target efficiency of Cas7-11 in our zebrafish experiments (~ 60–80% mRNA reduction) is comparable to that reported by Moreno-Sánchez et al. [21], its absence of detectable collateral cleavage positions its applications requiring transcriptome-wide integrity.

Notably, initial attempts to detect Cas7-11 protein in zebrafish embryos were unsuccessful until we incorporated *Xenopus* β-globin 5′ and 3′ UTRs flanking the *Cas7-11* coding sequence. This modification enabled detectable protein expression, likely due to the translation-enhancing properties of *Xenopus* UTRs, particularly in oocytes and early embryos [36].

Injection of *Cas7-11* mRNA provides transient expression in zebrafish embryos, with detectable protein present only between 6 and 24 h post-injection. The restricted temporal window limits the system’s applicability for targeting genes whose expression occurs later in development. Future efforts to extend the half-life of the Cas7-11 protein, potentially through fusion with stabilizing domains such as XTEN [37–39], may broaden the utility of this system in zebrafish. Despite these limitations, Cas7-11 demonstrated effective and specific RNA knockdown, highlighting its potential as a versatile tool for future applications, including therapeutic strategies against RNA virus-associated diseases such as COVID-19 [40].

## Conclusion

In summary, we evaluated the capacity of Cas7-11 to function as an mRNA–sgRNA system for gene knockdown in zebrafish embryos and tested its gene knockdown activity in hESCs. The consistent editing performance across these evolutionarily distant model systems highlights that Cas7-11 is a robust and versatile RNA-editing tool with great potential for broad applicability in biomedical research and therapeutic development. Given its programmable RNA-targeting capabilities and compatibility with adeno-associated virus (AAV) delivery, Cas7-11 holds promise for future gene therapy applications, particularly in the treatment of RNA-associated diseases.

## Materials and methods

### Zebrafish maintenance and embryo production

All experiments involving zebrafish are under the regulation of the Hong Kong Animals (Control of Experiments) Ordinance standards for the use of animals in experimentation and were approved by the Ethical Committees of the Chinese University of Hong Kong and the Hong Kong Special Administrative Region Government. Zebrafish wild-type strains were maintained and bred under standard conditions [1]. Wild-type zebrafish embryos were obtained through natural mating of zebrafish of mixed ages (3–12 months). The selection of mating pairs was random from a pool of 5 males and 5 females. Zebrafish embryos were staged in hours post-fertilization (hpf) [41]. The injected or controlled zebrafish embryos were incubated in 0.1% methyl blue and kept in a 28 ℃ incubator. 50–200 embryos were injected per experiment (depending on the needs of the experiment). Adult fish (AB strains) were kept at the CUHK animal holding core following standard procedures [22, 41] and research guidelines approved by the University AEEC [22, 41].

### mRNA, sgRNA, and plasmids generation

In the zebrafish embryo knockdown experiments, the HvsCas7-11 mammalian expression (pDF0317) plasmid was obtained from Addgene. The *Cas7-11* sequence was subcloned into the *pCS2 +* vector following a *Kozak* sequence and followed by the *FLAG* tag sequence using primers *Kozak-Cas7-11-2FLAG*-Fw and *Kozak-Cas7-11-2FLAG*-Re (Table S1), DNA amplification was made by Q5 High-Fidelity DNA Polymerase (NEB, M0491), under the process that one cycle of 98 ℃ for 30 seconds, 35 cycles of 98 ℃ for 30 seconds, 67 ℃ for 30 seconds, 72 ℃ for 2 minutes, and one cycle of 72 ℃ for 5 minutes. The PCR product was separated by electrophoresis and collected by gel cutting. The collected PCR fragments were purified using the FavorPrep GEL/PCR Purification Mini Kit (FAGCK 001–1) according to the manufacturer’s instructions. The final yield of the DNA fragments was around 200 ng for the ligation with the vector. *pCS2+* (2 µg) vector was digested by BamHI and ClaI endonuclease and ligated with the *Cas7-11* sequence DNA fragments by using the Gibson Assembly Kit (NEB, E2611) as we previously did [42]. Then, the *Kozak*-*Cas7-11-2FLAG* sequence was amplified by *Globin-Kozak-Cas7-11-2FLAG*-*Globin* Fw and *Globin-Kozak-Cas7-11-2FLAG*-*Globin* Re primers (Table S1) as previously. Then, it was subcloned into the pRN3P_T3_ABEmax_IVT plasmid (Addgene 171761) between the 5’ *Xenopus β-globin* and 3’ *Xenopus β-globin* sequence to enhance the translation efficiency of the *Cas7-11* sequence in zebrafish embryos [43]. The *pRN3P_T3_ABEmax_IVT* plasmid was digested by SmaI and NotI enzymes; the PCR fragment and digested vector were ligated using the Gibson assembly kit (NEB, E2611). The construct made above was detected as a codon-optimized plasmid (*5*′ *Xenopus β-globin*-*Kozak Cas7-11 2FLAG-3*′ *Xenopus β-globin*) in zebrafish. The zebrafish codon-optimized Cas7-11 plasmid was linearized with SfiI and transcribed in vitro using the T3 promoter Kit (Invitrogen), as we previously did [44]. The synthesized mRNA was then purified using the RNeasy Mini Kit (Qiagen). The concentration of *5*′ *Xenopus β-globin*-*Kozaq Cas7-11 2FLAG-3*′ *Xenopus β-globin* mRNA was detected by Nanodrop 2000 spectrophotometer (Thermo Scientific). The *EGFP* sequence was subcloned into a *pCS2 +* vector, the NotI enzyme linearized the *pCS2 + EGFP* plasmid, and *EGFP* mRNA was transcribed in vitro as we did previously by using the mMESSAGE mMACHINE SP6 Kit (Invitrogen) [42]. cDNA from zebrafish embryos was reverse-transcribed using RNA extracted at 6, 12, and 24 hpf (HiScript III 1st Strand cDNA Synthesis Kit, Vazyme). The *tbxta* ORF was PCR amplified from the cDNA by Q5 polymerase and *tbxta* CDS Fw and *tbxta* CDS Rev primers (Table S1). The *tbxta* ORF DNA fragment was amplified again by using the *Tbxta-2FLAG* Gibson Fw and *Tbxta-2FLAG* Gibson Rev primers. The *pCS2 + 2FLAG* vector was digested with EcoRI and Xbal, and the resulting backbone was assembled with the PCR-amplified insert by using the Gibson assembly enzyme kit (NEB, E2611). The resulting *pCS2 + 2FLAG-tbxta* plasmid was linearized with the NotI enzyme for in vitro transcription. mRNA of *tbxta-2×FLAG* was synthesized using the mMachine SP6 kit (Invitrogen). The concentration of *tbxta-2*×*FLAG* mRNA was detected by Thermo Scientific Nanodrop 2000. sgRNAs targeting *EGFP*, *tyrosinase*, and *tbxta* mRNA were designed according to CRISPR-Cas7-11 targeting data [19], in which there was no significant protospacer adjacent motif (PAM) reported, and sgRNA activity partially correlated with target accessibility [19]. In addition, the guide RNA sequences (22 nt) were analyzed using RNAfold software (http://rna.tbi.univie.ac.at/cgi-bin/RNAWebSuite/RNAfold.cgi). For all the Cas7-11 sgRNA transcription, oligonucleotides carrying the T7 promoter and appropriate downstream sequence were ordered from BGI and annealed with an antisense direct repeat oligo for sgRNA, and then the two oligos were PCR-amplified as DNA templates for in vitro transcription [19, 45]. The sgRNA was synthesized by the T7 Kit (Invitrogen) as we did previously [46]. A sgRNA universal oligo *Cas7-11*_*Universal_T7* (Table S2) containing the T7 promoter (5′-TAATACGACTCACTATA-3′) and the direct repeat of Cas7-11 (28 nt) preceded by 5’GG. A specific oligo, consistent with the spacer (22 nt for target-binding) and part of the sequence that is reverse complementary to the universal oligo. A 69 bp PCR product was generated following these conditions: 3 min at 95 ℃, 35 cycles of 30s at 95 ℃, 30s at 51 ℃, and 30s at 72 ℃, and a final step at 72 ℃ for 7 min. The PCR reaction was performed using Q5 polymerase. PCR products were purified using Favorprep Gel/PCR purification kit (Favorgen FAGCK001-1) columns and used as templates (100–300 ng) for T7 in vitro transcription (Invitrogen). Three sgRNAs were mixed, and the mixed sgRNA product was purified using the RNeasy Mini Kit (Qiagen 74204). sgRNA was visualized in a 2% agarose gel to check for RNA integrity and quantified using a NanoDrop 2000. To increase mRNA depletion, three sgRNAs targeting the same mRNA were co-injected.

In mammalian cells, the two oligos anneal to form a double-stranded PCR template (Table S3). Briefly, 10 µM oligo 1 and 10 µM oligo 2 were mixed with NEB buffer 2.1, generating the double-stranded oligo following these conditions: 95 ℃ for 2 min, with temperature decreasing at 1 ℃ per minute, to 25 ℃. The *pLentiCRISPR_mCherry* plasmid (addgene ID: 229078) was digested with the *BsmB*1 enzyme and ligated with the oligo using Solution1 (Takara 0621). The constructed plasmid was digested by XbaI and BamHI to insert *the Cas7-11 sequence* by using *Lenti-Discas711-mCherry*-Fw and *Lenti-Discas711-mCherry-*Rev primers (Table S1). The ligation of the oligo and digested vector was conducted using the Gibson Assembly Kit (NEB, E2611).

### Zebrafish embryo injection and image

Acquisition 2 nL containing 300 pg of *Cas7-11* mRNA and 600 pg of sgRNA mixture, injected into one-cell stage embryos (the injected components could be seen in the figure legend). The zebrafish embryo samples were collected for phenotype observation, protein, RNA extraction, and WISH during 6 h to 4 days post-injection. Zebrafish embryo phenotypes and fluorescent images were captured using a Nikon SMZ800 microscope.

### Whole-mount in *situ* hybridization (WISH) and *shh* RNA probe synthesis

WISH was performed using digoxigenin-labeled antisense RNA probes, as we previously did [42]. The RNA was extracted from zebrafish embryos (6 hpf, 12 hpf, 24hpf, and 48 hpf mixture) by TRIzol, and reverse transcribed into cDNA by HiScript III 1st Strand cDNA Synthesis Kit (Vazyme). *shh* was amplified by PCR using the primers *shh* CDS Fw and *shh* CDS Rev (Table S1). The *shh* probe Fw and *shh* probe Rev (Table S1) were used to amplify the *shh* CDS sequence for insertion into the *pBluescript+* vector. The *pBluescript + shh* plasmid was linearized by NotI enzyme and in vitro transcribed by T3 enzyme, which will synthesize an antisense mRNA of *shh*. (Invitrogen, mMESSAGE mMACHINE™ T3 transcription kit).

### qRT-PCR

Total RNA was isolated using the standard TRIzol protocol, as described in the manual (Thermo Fisher Scientific). The cDNA was synthesized using HiScript III 1st Strand cDNA Synthesis Kit (gDNA wiper) (Vazyme), following the manufacturer’s protocol. 2 µL of cDNA was mixed with 10 mM Forward and Reverse primers (0.2 µL) (Table S1) and 5 µL of ChamQ Universal SYBR qPCR Master Mix (Vazyme, Q711-02) in a 10 µL reaction. Then, run the system in the QuantStudio 7 Pro Flex Thermo Cycler (Applied Biosystems) under the following conditions: one cycle at 95 ℃ for 30 s, 35 cycles at 95 ℃ for 10 s, and at 60 ℃ for 34 s, and finally use the system collection feature provided by the machine.

### RNA-seq libraries and analysis

Total RNA was extracted using Trizol reagent according to the manufacturer’s instructions (Invitrogen). The cDNA libraries were then constructed using the SureSelect Strand Specific RNA Library Preparation Kit (Agilent) according to the manufacturer’s protocol.

RNA libraries for high-throughput sequencing were also constructed using the NEBNext Ultra RNA Library Prep Kit for Illumina (NEB, E7775L). Next-generation sequencing was performed by JMDNA (www.jmdna.com).

RNA-seq raw data were processed using the standard RNA-seq analysis pipeline. In brief, after checking the read quality with fastp (version 0.22.0), the reads were mapped to the Danio rerio genome (GRCz11) using HISAT2 (version 2.2.1), and reads with multiple alignments were excluded from downstream analysis. The output SAM file was then transformed into BAM format, sorted, indexed, and converted to count files (number of reads per transcript) using SAMTOOLS (version 0.1.18). The reads mapped to each gene were annotated using StringTie (version 2.2.3). Differential expression analyses were conducted on the basis of the edgeR package. Volcano plots were generated to visualize the significance of differentially expressed transcripts in different libraries using ggplot2 and custom scripts. Websites used in analysis:

fastp: https://github.com/OpenGene/fastp.

HISAT2: http://daehwankimlab.github.io/hisat2/manual/.

SAMTOOLS: https://www.htslib.org/doc/samtools.html.

StringTie: https://ccb.jhu.edu/software/stringtie/index.shtml.

edgeR: https://bioconductor.org/packages/release/bioc/html/edgeR.html.

### Adeno-associated virus (AAV) package

The Cas7-11 system was packaged into the AAV by co-transfection of 4.0 µg Cas7-11-sgRNA plasmids with 4.0 µg AAV8 capsid vector (addgene, 112864) and 8.0 µg AAV helper pAdDeltaF6 plasmid (addgene, 112867), using Lipomaster 2000 (Vazyme) into the HEK293FT cell (15 cm dish) according to the manufacturer’s instructions. Then, add the production into the cell culture medium gently. After 48 h post-transfection, the medium containing the loaded viral vector was filtered through a 0.45-mm filter (Sigma-Aldrich). The collected supernatant was concentrated using an Amicon Ultra-15 Centrifugal Filter Unit (MWCO 100 kDa) and then centrifuged at 16,000 g for 30 min. The concentrated virus was washed with DPBS, and then the final product was aliquoted and stored at -80 °C. The viral concentration was measured using Real-Time PCR methods (Figure S3). Based on the Ct values of the reference DNA. The stored concentration of the virus containing the Cas7-11 system was 2 × 10^12^ vg/ml, the working concentration was 2 × 10^9^ vg/ml (Figure S3).

### Cell culture and transfection

For human embryonic stem cells (H7), the cell culture dish should be coated with 1% Matrigel (Corning), usually in a 6 cm cell culture dish. Before transfection, the cells should be 70% confluent. Human embryonic stem cells are cultured in mTeSR™ complete medium (STEMCELL). The ratio of plasmid (in amount) to Lipofectamine Stem Transfection Reagent (Thermo Fisher, STEM00001) (in volume) is 1:4. Transfection was conducted according to the manufacturer’s instructions. For HEK293FT and HEK293T cells, the growth medium is 10% FBS in DEME (Thermo Fisher). The growth conditions are 37 ℃ and 5% CO_2_. The HEK293T cells were infected with the adeno-associated virus (AAV). In this study, both transfection and infection were carried out for 48 h.

### Western blot

The western blot was performed as described [42, 47]. Briefly, zebrafish embryos (*n* = 30 per sample) were washed with PBS and lysed in 200 µL of lysis buffer (137 mM NaCl, 5 mM EDTA, 10 mM Tris-HCl pH 7.5, 0.5% Triton X-100) supplemented with protease inhibitors using micropestles, followed by incubation on ice for 15 min. HEK293T cells were washed twice with PBS and lysed in the same buffer, then incubated on ice for 15 min. Both lysates were centrifuged at 15,000 g for 15 min at 4 °C, and the supernatants were collected. Protein samples were mixed with loading buffer, boiled at 100 °C for 5 min, and cooled to room temperature. Samples were resolved on a 10% SDS-PAGE gel. Proteins were transferred to a membrane using a Bio-Rad semi-dry apparatus at 15 V for 90–120 min. Primary antibodies, i.e., Anti-FLAG M2 (Sigma), Anti-Tyrosinase (Novus), Anti-GAPDH (Santa Cruz) at 1:3000 dilution, and Anti-beta Tubulin (Abcam) at 1:5000 dilution, were diluted in blocking buffer and incubated with the membrane overnight at 4 °C with shaking. After three 10-minute washes with TBST, the membrane was incubated with HRP-conjugated anti-mouse or anti-rabbit secondary antibodies (1:20,000 dilution in blocking buffer). Chemiluminescent signals were developed using ECL solution and captured with a Bio-Rad exposure system.

### Luciferase measurement

Cells treated with either plasmids or viral particles, as indicated, were cultured for 48 h before the supernatant and mRNA were collected. The Gaussia luciferase (GLuc) and Cypridina luciferase (CLuc) are secreted proteins, allowing them to be observed both intracellularly and extracellularly. A 0.45 μm filter was used to remove the cell debris and collect the supernatant. Then, the supernatant luciferase was detected using the UltraBriteTM Cypridina-Gaussia dual luciferase Kit (Targeting Systems, DLAR-4 SG-500), according to the manufacturer’s instructions. The cells were washed twice with PBS, then, lysed by the UltraBriteTM Cypridina-Gaussia dual luciferase assay reagent Kit and detected for luciferase intracellularly. The luciferase signal was measured using the Thermo Varioskan LUX Multi-mode Microplate Reader, with a read time of 1 s.

### Quantification and statistical analysis

GraphPad Prism 8 was utilized to assess the significance of the target gene in both the treatment, control, and blank groups. An unpaired Student’s t-test analysis was employed to determine significance among the various groups when comparing the RNA levels. A chi-square test was used to quantitatively analyze differences between rescue and knockdown phenotypes. All data were presented as mean ± standard error of the mean (SEM).

## Supplementary Information

Below is the link to the electronic supplementary material.

Supplementary file1 (docx 573 KB)

## Data Availability

All data supporting the findings of this study are available within the paper and its Supplementary Information. The datasets (RNA-Seq) analyzed in this study are available from the China National Center for Bioinformation (https://www.cncb.ac.cn/services). Primer sequences are provided in Supplementary Table (1) sgRNA sequences for zebrafish are provided in Supplementary Table (2) sgRNA sequences for hESCs are provided in Supplementary Table 3.

## Abbreviations

PBS: Phosphate-buffered saline
ddH_2_0: Double-distilled water
CRISPR: Clustered regularly interspaced short palindromic repeats
Cas: CRISPR-associated system
cdk2ap2: Cyclin-dependent kinase 2-associated protein 2
CLuc: Cypridina luciferase
DEPC: Diethyl pyrocarbonate
DEME: Dulbecco’s modified eagle medium
DR: Direct repeat
DPBS: Dulbecco’s phosphate-buffered saline
EGFP: Enhanced green Fluorescent Protein
FBS: Fetal bovine serum
GAPDH: glyceraldehyde-3-phosphate dehydrogenase
GLuc: Gaussia luciferase
taf15: TATA-box binding protein-associated factor 15
tbxta: T-box transcription factor Ta
LTR: Long terminal repeat
mRNA: message RNA
UTR: Untranslated region
OCT4: Octamer-binding transcription factor 4
SOX2: SRY-Box transcription Factor 2
TBST: Tris buffered saline with tween-20
PCR: Polymerase chain reaction
