# Supplementary material for: Programmable RNA targeting with clustered regularly interspaced short palindromic repeats (CRISPR) effector Cas7-11 in zebrafish embryos and mammalian cells
Source: Cell Biosci. 2026 Aug 17;16:123. doi: 10.1186/s13578-026-01632-w (PMC13757028; doi:10.1186/s13578-026-01632-w)
Supplement: Supplementary file 1 [file 13578_2026_1632_MOESM1_ESM.docx]

## **Figure S1**

**Figure S1. Optimization of Cas7-11 expression in HEK293T cells and zebrafish embryos. (A)** Schematic diagram of different designs with *Cas7-11* sequence was fused with a 2×*FLAG* sequence. The HEK293T cells seeded in a 6-well plate were transfected using 1 μg of plasmids 1-4 as indicated. After 48 hours, cells were lysed and probed with FLAG and GAPDH antibodies. The Cas7-11-2FLAG protein in the No.2 version (*pCS2-Kozak-Cas7-11-2FLAG*) showed an increased expression level compared to the other three groups. **(B)** The Cas7-11-2FLAG protein in the No.2 version (*pCS2-Kozak-Cas7-11-2FLAG*) showed no signal in zebrafish embryos without *Xenopus globin* *5'-UTR* and *3'-UTR* sequences on both sides of the *Cas7-11* sequence.

## **Figure S2**

**Figure S2.** **A linear relationship exists between the Ct values and the viral titers.** The concentration of virus containing *Cas7-11-sgGLuc* was 2.13×10^12^ vg/ml, and virus containing *Cas7-11-sgNT* was 2×10^12^ vg/ml. Equation calculated by the linearity based on the density gradient of the *Cas7-11* plasmid from 10 ng to 1×10^-4^ ng/μl, which is equivalent to 2.13×10^9^ to 2.13×10^4^ copies of the virus. R^2^=0.9804 in the *Cas7-11-sgGLuc* group and 0.9873 in the *Cas7-11-sgNT* group. The value fell within the confidence interval.

## **Figure S3**

**Figure S3. qPCR validation of candidate genes identified by RNA-seq.** qPCR analysis confirmed that the candidate genes showing no significant changes in the RNA-seq volcano plot were not differentially expressed between the experimental and control groups. Data are presented as mean ± SEM from three independent biological replicates (n=20 embryos per replicate). *cdk2ap* mRNA was used as the internal normalization control. n.s., not significant (Student's t-test).

# **Supplementary information**

## Table S1. Primers for The Construction of Plasmids and Quantitative Real-Time PCR

| **Table S1 Primers for The Construction of Plasmids and Quantitative Real-Time PCR** | |
| --- | --- |
| Name | Sequences (5’→3’) |
| *Tbxta-2FLAG* Gibson Fw | ACAAGGATGATGATGATAAGATGTCTGCCTCAAGTCCC |
| *Tbxta-2FLAG* Gibson Re | TAATACGACTCACTATAGTTTCAGTAGCTCTGAGCCAC |
| *tbxta* *CDS* Fw | ATGTCTGCCTCAAGTCCCGA |
| *tbxta* *CDS* Re | TGAAGCGATCTCAGTAGCTCTG |
| *tyr* Q-PCR Fw | TCCCCAGAAGTCCTCCAGTC |
| *tyr* Q-PCR Re | TGACAGAACCCTCGACCTGA |
| *EGFP* Q-PCR Fw1 | GGCAAGCTGACCCTGAAGTT |
| *EGFP* Q-PCR Re1 | GGCGGACTTGAAGAAGTCGT |
| *EGFP* Q-PCR Fw2 | GGCATCGACTTCAAGGAGGA |
| *EGFP* Q-PCR Re2 | GCTCAGGTAGTGGTTGTCGG |
| *tbxta* Q-PCR Fw1 | CGCAAGACTTCCGGAAGAGT |
| *tbxta*_Q-PCR Re1 | GAACCACAGAGCTGCTCCAT |
| *tbxta* Q-PCR FW 2 | ACCAAGACTGGGAGACGAATGT |
| *tbxta* Q-PCR Re2 | ACCGTTCACGTATTTCCACCGA |
| *cdk2ap2* Q-PCR Fw | AGGATCTTGTGGCTCTTCTCCATCAC |
| *cdk2ap2* Q-PCR Re | TTTCACGGCTCATCTCCTCAATGAC |
| *taf15* Q-PCR Fw | GCTGCAGCATTGAAAACCTTCAGTC |
| *taf15* Q-PCR Re | TCCATCACACACACACACTAAACGC |
| *Kozak-Cas7-11-2FLAG*-Fw | GCTACTTGTTCTTTTTGCAGGCCGCCATGGTCAAGTCCATCCCTATAACACTGAC |
| *Kozak-Cas7-11-2FLAG*-Re | TGTAATCCATAGCTTTAAATTTAGAGGCCACAATCCAGCG |
| *Globin-Kozak-Cas7-11-2FLAG*-*Globin* Fw | GATCTGAATTCCTGCAGCCCGCCGCCATGGTCAAGTCC |
| *Globin-Kozak-Cas7-11-2FLAG*-*Globin* Re | TTAGTGGTAACCAGATCCGCCTATAGTTCTAGAGGCTCGAGAGG |
| AAV-*Cas7-11-sgNT-2FLAG-*Fw | CAGAAGAAGCTGACCACGCCAGATTACAAGGATGATGATGATAAG |
| AAV-*Cas7-11-sgNT-2FLAG*-Re | ATGCCCACGGTGTCCACGGCCTATAGTTCTAGAGGCTCG |
| AAV-*Cas7-11-sgGLuc-2FLAG-*Fw | CAGAAGAAGCTGACCACGCCAGATTACAAGGATGATGATGATAAG |
| AAV-*Cas7-11-sgGLuc-2FLAG*-Re | ATGCCCACGGTGTCCACGGCCTATAGTTCTAGAGGCTCG |
| *EFS* Promoter Q-PCR Fw1 | GGGGTAAACTGGGAAAGTGATGTCGT |
| *EFS* Promoter Q-PCR Re1 | CTGTGTTCTGGCGGCAAACC |
| *EFS* Promoter Q-PCR Fw2 | CGTTCTTTTTCGCAACGGGT |
| *EFS* Promoter Q-PCR Re2 | ATCTTGCAAAAGCTTGGCCG |
| *GAPDH* (Human) Q-PCR Fw | GTCTCCTCTGACTTCAACAGCG |
| *GAPDH* (Human) Q-PCR Re | ACCACCCTGTTGCTGTAGCCAA |
| *shh* CDS Fw | ATGCGGCTTTTGACGAGAGTGCTG |
| *shh* CDS Re | TAATACGACTCACTATAGGGTCAGCTTGAGTTTACTGACATC |
| *shh* probe Fw | CCACCGCGGTGGCGGCCGCTATGCGGCTTTTGACGAGAG |
| *shh* probe Re | TATCGATAAGCTTGATATCGTCAGCTTGAGTTTACTGACATC |
| AAV-*Cas7-11* Q-PCR Fw | GCCCGACTTGGAGTATACCG |
| AAV-*Cas7-11* Q-PCR Re | TCCGCCATTGCTTCCAATCT |
| *Lenti-Discas711-2FLAG-puro*-Fw | ACCGTGTTTGAAGGAGGATAATAAGGTTGATGTTGGAACATGTAAACCATGTAGTTGAGGTCA |
| *Lenti-Discas711-2FLAG-puro*-Re | GAGAGAAGTTTGTTGCGCCGTTTAGTTCTAGAGGCTCGAG |
| *Lenti-Discas711-mCherry*-Fw | GCCAGAACACAGGACCGGTTATGACGACTACTATGAAGATTTCAATTGAATTCCTCGAG |
| *Lenti-Discas711-mCherry*-Re | GAGAGAAGTTTGTTGCGCCGTTTGCCCACGGTGTCCACGG |
| *Puro*-colony PCR Fw | ACCGGTTGATGTCACGGAAC |
| *Puro*-colony PCR Re | GCAGGTTTTCCGCGCTTCTA |
| *CLuc* Q-PCR Fw | GTTGGGGGTCAGATCACAGG |
| *CLuc* Q-PCR Re | AAGCAACTGCTCGTCGTACA |
| *GLuc* Q-PCR Fw | CTGCCTGTCCCACATCAAGT |
| *GLuc* Q-PCR Re | TGTGCGATGAACTGCTCCAT |
| *OCT4* Q-PCR Fw | CCTGAAGCAGAAGAGGATCACC |
| *OCT4* Q-PCR Re | AAAGCGGCAGATGGTCGTTTGG |
| *SOX2* Q-PCR Fw | GCTACAGCATGATGCAGGACCA |
| *SOX2* Q-PCR R | TCTGCGAGCTGGTCATGGAGTT |
| chchd4a_F | CGTACTGCAAGCAGGAAGGTAA |
| chchd4a_R | AAACACGGGCAGTTCCAGTT |
| Hexa_F | AACATAACGGCCGCTCTCAA |
| Hexa_R | GTGCCCTGTGGAATTCGTTC |
| Cep89-F | TCCCTTGAGAGACAGAGCAGA |
| Cep89_R | GCAATGATGATGGCTGCGTT |
| cox19_F | GAGTGCAGAATGGACAGACAGT |
| cox19_R | AAGCATGGGCTCTGGCTAAT |
| cog1_F | CCAGCAGTCAGATCCGGTTT |
| cog1_R | TTGAGGAGGGAGCCAACAGT |
| dhx40_F | AGCAGTGAAACAGAGCACCA |
| dhx40_R | CCATTCCTGCTAAGACCTGCT |
| ube2b_F | CGCTGTCATTTTTGGGCCTG |
| ube2b_R | GCGGCTTGTTGGGATACTCT |

## Table S2. Zebrafish Embryo Targeting Oligo Sequences

| **Table S2 Zebrafish Embryo Targeting Oligo Sequences** | |
| --- | --- |
| Name | Sequences (5’→3’) |
| *Cas7-11_tbxta1* | TCAGAGCCAGTGTCACCGGTCTACATCAACCTTATTATCCTCCTTCAAAC |
| *Cas7-11_tbxta2* | TGGTCAGCCCTGCAGATCCCCAACATCAACCTTATTATCCTCCTTCAAAC |
| *Cas7-11_tbxta3* | GTCAGTTGCAGGGACGACTCTCACATCAACCTTATTATCCTCCTTCAAAC |
| *Cas7-11_tbxta4* | GCACCCGTATCTTTCAGCAAAGACATCAACCTTATTATCCTCCTTCAAAC |
| *Cas7-11_tbxta5* | GTGAGAGATACTCCAGCTTGAGACATCAACCTTATTATCCTCCTTCAAAC |
| *Cas7-11_tbxta6* | TCGGTCCTGCTGGATTTTGTGGACATCAACCTTATTATCCTCCTTCAAAC |
| *Cas7-11_tyrosinase1* | CCGGCGACGGCTCCGTGTGCGGACATCAACCTTATTATCCTCCTTCAAAC |
| *Cas7-11_tyrosinase2* | AAACCTGCCAGTGCGCCGGAAAACATCAACCTTATTATCCTCCTTCAAAC |
| *Cas7-11_tyrosinase3* | GTGACGGGTCGCAGTTTGATGCACATCAACCTTATTATCCTCCTTCAAAC |
| *Cas7-11_tyrosinase4* | CTCCTCCTCTTCTTCTTCCTCCACATCAACCTTATTATCCTCCTTCAAAC |
| *Cas7-11_tyrosinase5* | CACTACCCCACCGCCAACGCCCACATCAACCTTATTATCCTCCTTCAAAC |
| *Cas7-11_tyrosinase6* | TTATAATTTATTCTACGTTAAAACATCAACCTTATTATCCTCCTTCAAAC |
| *Cas7-11_EGFP1* | GCTGTTCACCGGGGTGGTGCCCACATCAACCTTATTATCCTCCTTCAAAC |
| *Cas7-11_EGFP2* | CGTGCCCTGGCCCACCCTCGTGACATCAACCTTATTATCCTCCTTCAAAC |
| *Cas7-11_EGFP3* | GCATCGACTTCAAGGAGGACGGACATCAACCTTATTATCCTCCTTCAAAC |
| *Cas7-11_NT* | CAGACTATGCGTCGACAAGCCACATCAACCTTATTATCCTCCTTCAAAC |
| *Cas7-11_ntl7* | GGACTGATGCTGCAGTTATGGAACATCAACCTTATTATCCTCCTTCAAAC |
| *Cas7-11_ntl8* | AGAATGTAACAGTCTTACATGTACATCAACCTTATTATCCTCCTTCAAAC |
| *Cas7-11_ntl9* | ATATCTATCTATTTGTATTTGTACATCAACCTTATTATCCTCCTTCAAAC |
| *Cas7-11*_Universal_T7 | TAATACGACTCACTATAGGGTTTGAAGGAGGATAATAAGGTTGATGT |

## Table S3. Human Embryonic Stem Cells Targeting Oligo Sequences

| **Table S3 Human Embryonic Stem Cells Targeting Oligo Sequences** | |
| --- | --- |
| Name | Sequences (5’→3’) |
| sg*SOX2* Oligo1 | CACCGGTTGATGTCACGGAACCCCTGACAGCCCCCGTCACA |
| sg*SOX2* Oligo2 | AAACTGTGACGGGGGCTGTCAGGGGTTCCGTGACATCAACC |
| sg*OCT4* Oligo1 | CACCGGTTGATGTCACGGAACGGTGAAATGAGGGCTTGCGAAGGGACTACT |
| sg*OCT4* Oligo2 | AAACAGTAGTCCCTTCGCAAGCCCTCATTTCACCGTTCCGTGACATCAACC |
| sg*NT* Oligo1 | CACCGGTTGATGTCACGGAACGGTAATGCCTGGCTTGTCGACGCATAGTCTG |
| sg*NT* Oligo2 | AAACCAGACTATGCGTCGACAAGCCAGGCATTACCGTTCCGTGACATCAACC |
